# Epigenetic Alterations and Biomarkers for Immune Checkpoint Inhibitors–Current Standards and Future Perspectives in Malignant Pleural Mesothelioma Treatment

**DOI:** 10.3389/fonc.2020.554570

**Published:** 2020-12-14

**Authors:** Yoshie Yoshikawa, Kozo Kuribayashi, Toshiyuki Minami, Masaki Ohmuraya, Takashi Kijima

**Affiliations:** ^1^ Department of Genetics, Hyogo College of Medicine, Nishinomiya, Japan; ^2^ Department of Respiratory Medicine and Hematology, Hyogo College of Medicine, Nishinomiya, Japan

**Keywords:** chromatin modification, histone methylation, asbestos, mutations, immunotherapy

## Abstract

Malignant pleural mesothelioma (MPM) is strongly associated with occupational or environmental asbestos exposure and arises from neoplastic transformation of mesothelial cells in the pleural cavity. The only standard initial treatment for unresectable MPM is combination chemotherapy with cisplatin (CDDP) and pemetrexed (PEM). Further, CDDP/PEM is the only approved regimen with evidence of prolonged overall survival (OS), although the median OS for patients treated with this regimen is only 12 months after diagnosis. Thus, the development of new therapeutic strategies has been investigated for approximately 20 years. In contrast to recent advances in personalized lung cancer therapies, diagnostic and prognostic biomarker research has just started in mesothelioma. Epigenetic alterations include DNA methylation, histone modifications, and other chromatin-remodeling events. These processes are involved in numerous cellular processes including differentiation, development, and tumorigenesis. Epigenetic modifications play an important role in gene expression and regulation related to malignant MPM phenotypes and histological subtypes. An immune checkpoint PD-1 inhibitor, nivolumab, was approved as second-line therapy for patients who had failed initial chemotherapy, based on the results of the MERIT study. Various clinical immunotherapy trials are ongoing in patients with advanced MPM. In this review, we describe recent knowledge on epigenetic alterations, which might identify candidate therapeutic targets and immunotherapeutic regimens under development for MPM.

## Introduction

Malignant pleural mesothelioma (MPM) remains one of the most incurable malignancies, with a very poor prognosis and a 7.9-month median survival time (MST) from diagnosis to death (1). Even after the widespread prevention of asbestos use, mortality rates have not decreased substantially in most countries. Further, the use of asbestos is not banned worldwide, and is still collected and used in many countries (e.g., China, Russia, and India) (2). Thus, the incidence of mesothelioma is not expected to peak before 2020 because asbestos was widely used in most developed countries until 1970 and the long latency (about 30 to 50 years) of asbestos exposure (3).

When asbestos fibers reach the lung periphery and the pleura, they cause local chronic inflammation and carcinogenesis. Common markers of carcinogenic processes include overexpression of growth factors such as VEGF, inactivating mutations of tumor suppressor genes (*BAP1*, *CDKN2A*, *NF2*, and *TP53*), extensive chromosomal deletions, and epigenetic alterations, which are essential for genetic abnormalities in malignant mesothelioma (4) (**Table 1**). These factors result in enhanced cell proliferation, apoptosis resistance, and local tumor immune evasion, and are the targets of several new therapeutic approaches (16).

**Table 1:** Summary of the biomarkers for diagnosis or as therapeutic targets for malignant mesothelioma.

Alteration	Process	Phenotypes and associated molecules	Characteristics	References
Genomic	Sequence-level mutation	Common mutated genes *BAP1*, *NF2*, *TP53*, *SETD2*, *SETDB1*	Low mutation rate (<2 somatic mutations per megabase of exons)	5–7
	Copy number alteration	Frequent losses in 9p21.3 (*CDKN2A*), 3p21.1 (*BAP1, SETD2, PBRM1*), 22q12.2 (*NF2*), 13q12.11 (*LATS2*), 6q25.1 (*LATS1*), 16p13.3 (*RBFOX1*)	Complicated chromosomal rearrangements, such as chromothripsis or chromoplexy, causing fusion transcripts and neoantigen expression	5, 7–9
Epigenetic	DNA methylation	Overexpression of DNA methyltransferases *DNMT1*, *DNMT3A*, and *DNMT3B*	Level of DNA methylation associated with histological types and prognosis	7, 10, 11
	Histone modifications	Functional loss of deubiquitinating enzyme, BAP1, and histone methyltransferases, SETD2 and SETDB1. Overexpression of *EZH2* and *SUZ12*	Dysregulated polycomb gene expression contributing the pathogenesis	5, 10, 12
	Chromatin remodeling	Frequent mutations of SWI/SNF complex genes including PBRM1	Development of anti-cancer drugs using synthetic lethality	8, 13
	Non-coding RNAs	Global suppression of miRNA expression. lncRNAs associated with prognosis	miRNAs as circulating biomarkers	7, 14, 15

Immune mechanisms may be important therapeutic targets in MPM, as demonstrated by tumor-infiltrating lymphocytes (17, 18), spontaneous regression of mesothelioma (19, 20), and tumor responses to immunotherapy (21). Indeed, immunotherapy with immune checkpoint inhibitors (ICIs) has dramatically improved the prognosis of various solid tumor types, including melanoma and non-small cell lung cancer (22). Several clinical trials show that ICIs may also have promising antitumor effects in MPM (23).

Conversely, no validated biomarker is currently available for these agents, and even the representative biomarker PD-L1 has limited significance. In fact, recent clinical trials have shown that the role of ICIs in MPM is still debated, especially when considering single agent use (24).

## Mutations

Recently, various gene mutations were identified in MPM *via* technological advances in genetic analysis. However, unlike other cancer types, active genetic mutation types such as epidermal growth factor receptor (EGFR) mutations in lung adenocarcinoma are not well understood in MPM. However, inactivating mutations of multiple tumor suppressor genes have been identified (5).

The most frequent tumor suppressor gene mutation in MPM is an inactivating mutation of *CDKN2A* (cyclin-dependent kinase inhibitor 2A gene), which occurs in approximately 70% or more MPM cases (25). *CDKN2A* has 3 exons and codes for 2 proteins: pl6/INK4a and p14/ARF.

pl6/INK4a is a cyclin-dependent kinase (CDK) inhibitor that inhibits pRB phosphorylation by CDK 4/6-cyclin D and arrests the cell cycle at G_1_. p14/ARF inhibits p53 degradation by MDM2 activity and stabilizes p53 function. When inactivating *CDKN2A* mutations render the pl6 and pl4 proteins nonfunctional, pRB and p53 lose their ability to regulate the cell cycle, leading to constitutive cell growth. Thus, CDKN2A is strongly associated with MPM development (6).

Neurofibromin2 (*NF2*) is a tumor suppressor gene located on the long arm of chromosome 22. Inactivating mutations in *NF2* are reported in approximately 40-50% of MPMs (26). *NF2* encodes the Merlin protein, and deletion of the Merlin protein results in constant yes-associated protein (YAP) activation by Hippo pathway inactivation (26, 27). YAP controls cell proliferation, survival, differentiation, organ size maintenance, tissue homeostasis, and plays a central role in the Hippo pathway (28). Merlin negatively regulates mTOR, and deletion of Merlin leads to mTOR signaling activation (29).

BRCA1-associated protein 1 (*BAP1*) is a tumor suppressor gene located on the short arm of chromosome 3, and the overall frequency of *BAP1* alterations, including single-nucleotide variants, small indels, several exons to whole gene deletions, and structural variants, is reported to be approximately 57% in MPMs (7, 30). BAP1 encodes deubiquitinating enzymes and is involved in the regulation of expression and transcription of many genes. Further, BAP1 is involved in DNA repair, especially double-strand breaks (31). Germline mutations in *BAP1* are reported in familial intraocular/cutaneous malignant melanoma. Similar mutations occur in familial MPM (32), drawing attention to the relationship between BAP1 and MPM.

Complicated chromosomal rearrangements such as chromothripsis and chromoplexy are two of the most characteristic genome alterations. Multiple noncontiguous minute deletions in 3p21 carrying *BAP1*, *SETD2*, and *PBRM1* (8) and fusion transcripts caused by gene fusions including 3p21 region and *NF2* have been reported (5). These chromosomal rearrangements produce neoantigen expression (9).

In a study, 2.4% of mesothelioma patients were diagnosed with microsatellite instability based on whole exome sequencing data, which revealed somatic mutations in the repair genes *MSH2*, *MSH6*, *MLH1*, *PMS2*, *EXO1*, *POLD1*, and *POLE* (33). Another study reported DNA mismatch repair (MMR) protein expression in all 159 MPM samples analyzed using IHC (34). Therefore, further studies on MMR molecules are warranted.

## DNA Methylation

Epigenetic modifications cause phenotypic changes that do not involve alterations in the DNA sequence. These changes significantly affect gene regulation and expression as they occur in various cellular processes including differentiation, development, and tumorigenesis. Epigenetic mechanisms include DNA methylation, histone modifications, and other chromatin-remodeling events. Non-coding RNAs also work as regulators in epigenetics.

DNA methylation is the main epigenetic modulation. Methylation occurs naturally on cytosine bases at CpG sequences. Clusters of CpG dinucleotides (CpG islands) (35) are located in promoters of approximately 60% of genes, and additional CpG dinucleotides are dispersed throughout the genome. The former mostly are unmethylated, and the latter are typically hypermethylated in normal cells (36, 37). DNA methyltransferases, including DNMT1, 3A, and 3B, mediate transfer of a methyl group from S-adenosyl-methionine to the 5’ position of cytosine at CpG dinucleotides. Methylation in CpG islands inhibits binding of methylation-sensitive transcription factors and silences genes. Indeed, transcriptionally silencing tumor suppressor genes causes tumorigenesis. Genome-wide DNA hypomethylation increases chromosomal fragility (36, 37).

Studies analyzing DNA methylation indicate that silencing tumor suppressor genes through site-specific DNA hypermethylation and genome-wide hypomethylation are observed in MPM. Goto et al. examined the methylation status of 6,157 CpG islands using methylated CpG island amplification-microarray in 20 MPM cases and compared them to 20 pulmonary adenocarcinomas (38). Approximately 387 genes (6.3%) were hypermethylated in MPM in comparison with 544 genes (8.8%) in lung adenocarcinomas. These results show that epithelial-type MPM with less methylation tends to have longer survival rates. Hypermethylation of three genes (*TMEM30B*, *KAZALD1*, and *MAPK13*) distinguish MPM from adenocarcinoma. Christensen et al. identified significant differences among the epigenetic profiles of MPM compared to normal pleura (39). Methylation of 1505 CpG loci associated with 803 cancer-related genes was studied in 158 MPM cases and 18 normal pleura samples using methylation arrays. This report showed that methylation status is significantly related with asbestos body burden and patient survival.

To develop the non-invasive methods for diagnosis, circulating biomarkers can be detected in liquid samples such as plasma, serum, or urine using a method that has attracted considerable attention. Guarrera et al. analyzed DNA methylation levels in the whole blood in 163 MPM cases and 137 non-tumor controls using a methylation array (40). Differential methylation levels between MPMs and controls were observed mainly in the immune system–related genes. The gene showing the most hypomethylated single-CpG was *FOXK1*.

TCGA data suggest that *DNMT1*, *DNMT3A*, and *DNMT3B* overexpression is correlated with shorter MPM patient survival (10). Blum et al. applied “a deconvolution approach” to extract the molecular profile characterizing epithelioid and sarcomatoid MPM (11). They integrated epigenetic data and showed a correlation of gene expression levels with their DNA methylation levels. Furthermore, the methylation level of CpG sites is associated with histology: the differentially methylated CpG sites associated with epithelioid or sarcomatoid MPM are non-CpG islands and CpG islands, respectively.

Clinical efforts made previously to restrain *DNMT* gene activity in MPM have been unsuccessful. Yogelzang et al. (41) reported a 17% overall response rate in 41 MPM patients who received 120-h continuous infusion of dihydro-5-azacytidine, a DNA methyltransferase inhibitor. It is interesting to note that one of the complete responders was free from disease for six years after treatment.

## Histone Modifications

Gene expression is controlled by loosening and compacting DNA wrapped around core histones (H2A, H2B, H3, and H4) through histone modification, three major types of which are known. Acetylation/deacetylation, methylation/demethylation, and ubiquitination/deubiquitination are major modifications in normal and cancer cells. Covalent modification is carried out at lysine-rich tails of core histones that extend out from the nucleosome. Histone acetylation increases the negative charge of the histone and resultantly repulses DNA, thus activating gene expression (42). Histone lysine methylation can activate or repress gene expression at some sites. Histone H3K9 (histone H3 at lysine 9) and H3K27 methylation are associated with transcriptional repression, while H3K4, H3K36, or H3K79 methylations are linked with gene activation (43). Histone H2A monoubiquitination is frequently correlated with gene silencing, while H2B monoubiquitination is mostly associated with transcriptional activation (44).

Lysine acetylation is catalyzed by histone acetyltransferase (HAT), which transfers the acetyl group from acetyl-CoA to the epsilon-amino group of a core histone protein lysine residue (45). Histone deacetylases (HDACs) are either Zn^2+^-dependent histone deacetylases or NAD+-dependent sirtuin deacetylases, which remove acetyl groups (46). Histone lysine methylation is mediated by numerous enzymes called histone lysine methyltransferase (KMTs) that mediate mono-, di-, and trimethylation of specific residues of lysine. On the contrary, histone demethylation is mediated by histone demethylases (KDMs) (43). Ubiquitin (Ub) conjugation reactions are catalyzed by the sequential action of Ub-activating (E1), Ub-conjugating (E2), and Ub-ligating (E3) enzymes. Deubiquitinating enzymes (DUBs) catalyze Ub removal from targeted substrates (44). E3 ubiquitin ligases, a large protein family, and DUBs have been well known as important regulators of numerous cellular processes (47).

*BAP1* encodes DUB, and it is one of the most frequently altered genes in MPM (5, 48, 49). BAP1 assembles DUB complexes with the transcription regulators Additional Sex Combs-like (ASXL1, ASXL2, and ASXL3) similar to the *Drosophila* ortholog Calypso binding to the Polycomb group protein, ASX (50). These proteins form the Polycomb repressive deubiquitinase (PR-DUB) complex that cleaves Ub from histone H2A in nucleosomes. PR-DUB activity is essential for the regulation of H2A ubiquitination and Polycomb group-dependent gene silencing.

Polycomb repressive complex 2, formed by EZH2, SUZ12, and EED protein subunits, induces gene silencing through the addition of methyl groups to histone H3 at lysine 27, leading to trimethylated histone H3 lysine 27 (H3K27me3) (51, 52). EZH2 and SUZ12 overexpression were identified in asbestos-associated MPM cell lines (10), and EZH2 was overexpressed in approximately 85% of MPMs compared to normal pleura (52). Gene expression of *EZH2* and *SUZ12* is associated with MPM patient survival (10). LaFave et al. reported that BAP1 loss in mice results in increased H3K27me3 and EZH2 expression (12). Additionally, mesothelioma cells that lack BAP1 are sensitive to the inhibitor EZH2. This finding could provide a novel therapeutic approach against malignancies with *BAP1* mutation.

The gene *SETD2* encodes a member of the SET domain family, which are histone methyltransferases specific for H3K36. This gene is frequently mutated in MPM (5, 8). In addition to *SETD2*, Bueno et al. observed somatic mutations of the histone methyltransferase family genes *SETDB1*, *SETD5*, *ASH1L*, *PRDM2*, and *KMT2D*, the histone demethylase gene *KDM2B*, and the histone acetyltransferase gene *CREBBP* in MPMs (5).

HDAC inhibitors have emerged as anti-cancer drugs to treat hematological and solid malignancies. They could lead to cancer cell cycle arrest, differentiation, cell death, reduce angiogenesis, and modulate immune responses (46). However, these drugs have never provided sufficient therapeutic effects in MPM with the use of a single agent. Krug et al. randomized 661 patients to receive either vorinostat (n=329) or placebo (n=332). In this trial, vorinostat given as a second- or third-line therapy did not improve OS [30.7 weeks (95% CI: 26.7–36.1) compared to 27.1 weeks (95% CI: 23.1–31.9) for patients receiving placebo]. Thus, vorinostat would not be suitable as a therapy for patients with advanced MPM (53).

## Chromatin Remodeling

Chromatin remodeling complexes are evolutionarily conserved multi-unit protein complexes that involve four subfamilies of ATP-dependent nucleosome-remodeling complexes: switch/sucrose non-fermentable (SWI/SNF), imitation switch, chromodomain helicase DNA-binding, and chromatin-remodeling ATPase INO80 (54). These complexes regulate chromatin structure to enable specific interactions with particular transcriptional activators, repressors, and histone modifiers. The SWI/SNF complex is the most well characterized tumor suppressor (55). Mammalian SWI/SNF complexes are composed of 12–15 subunits, and mutations in subunit genes including *ARID1A*, *SMARCA4*, *ARID1B*, *ARID2*, and *PBRM1* occur across a wide spectrum of human cancers (56). In MPM, we found frequent multiple minute simultaneous biallelic deletions on chromosome 3p21. The top four frequently altered genes are associated with epigenetic modifications: *BAP1*, *SETD2*, *PBRM1*, and *SMARCC1* (8). The last two genes belong to SWI/SNF complexes. We also detected sequence-level somatic mutations in *ARID2* and *SMARCA4* (13). The functional loss of these genes may lead to chromosomal instability because of their role in transcriptional regulation, DNA repair, and regulation of chromatin architecture and topology (57).

Synthetic lethality refers to cellular or organismal death that occurs when two genes are simultaneously perturbed (58). This phenomenon might be useful against tumor suppressor gene products in cancer-drug discovery. *ARID1A*, a SWI/SNF complex subunit, is frequently mutated in cancer. There are two mammalian homologs with an ARID domain, ARID1B and ARID2. Loss of ARID1B in ARID1A-deficient backgrounds destabilizes SWI/SNF complexes and impairs cancer and primary cell proliferation (59). This could be a conserved function underlying the synthetic lethality between ARID1A and ARID1B. Further, this finding increases information about synthetic lethality among chromatin remodeling subunits (60). To develop a new generation of anti-cancer drugs for MPM, synthetic lethality could be an attractive research field.

## NON-CODING RNAs IMMUNOTHERAPY WITH ICIs IN MPM

Non-coding RNAs are a cluster of RNAs that are not translated into a protein and play an important role in post-transcriptional gene silencing. Based on their size, RNAs are characterized into short chain non-coding RNAs, including siRNA, miRNA, and piRNA, and long non-coding RNAs (lncRNAs) (61). There is a global suppression of miRNA expression in MPM: for example, let-7 family, miR-31, miR-34 family, including miR-34b and miR-34c, and miR-15 family, including miR-15a and miR-16. Lo Russo et al. reviewed the biological roles of miRNAs in MPM and listed miRNAs with diagnostic and/or prognostic value (14). Numerous cell functions such as apoptosis, cell proliferation, migration, invasion, and cell death are modulated by miRNA. As regulators of biological reactions, the response of MPM toward platinum-based chemotherapy is strongly affected by miRNAs and lncRNAs (62). It is reported that increased programmed death-ligand 1 (PD-L1) expression is associated with downregulated miRNAs, including miR-15b, miR-16, miR-193a-3p, miR-195, and miR-200c (63). As therapeutic targets, the phase I clinical trial (NCT02369198) using TargomiRs, delivering an miR-16-based miRNA mimic in the minicell-based formulation, was performed, and acceptable safety was observed. This trial showed a partial response in 1 of 22 patients (64). It is increasing the probability of non-coding RNAs in serum or plasma as circulating MPM biomarkers (15). Downregulated expression of miR-126 in serum from MPM patients was confirmed by several groups, but the discrimination power between MPM patients and healthy controls is not so high (15). A combination of circulating biomarkers is expected to overcome the poor sensitivity and specificity of single markers.

The biomarkers or therapeutic targets described above are summarized in **Table 1**. Recent insights about epigenetic mechanisms and dysregulated gene expression in MPM provide new opportunities with therapeutically challenging MPM.

## Systemic MPM Treatment

To date, only cisplatin-pemetrexed combination therapy has been approved as frontline chemotherapy for unresectable MPM. This treatment was established as the standard of care in 2003, with no new modality (65). Platinum doublets, including pemetrexed, have been the international standard of care for MPM for approximately 20 years as first-line chemotherapy (66). However, the median OS for pemetrexed-cisplatin combination therapy does not exceed 13–16 months, according to data from randomized phase 3 trials (67). Thus, improved medical treatment is essential for improved outcomes.

## Immunotherapy With ICIs in MPM

Since the introduction of ICIs, immunotherapy has made dramatic advances in various cancers. There are now three clinically available ICIs that block each of the following immunosuppressive molecules: cytotoxic T-lymphocyte-associated protein 4 (CTLA-4), programmed death-1 (PD-1), and PD-1 ligand-1 (PD-L1) (68). However, the PD-L1 positivity ratio and tumor proportion score (TPS) are 20%–40% in MPM. PD-L1 binds and inhibits PD-1 on T cells. Further, PD-L1 is a spontaneously occurring factor that diminishes the anti-tumor immune response, and its expression is associated with poor prognosis in mesothelioma (69). The median OS of patients who are PD-L1-positive is 5.0 months compared to 14.5 months in patients who are PD-L1-negative. Thus, PD-L1 positivity is an independent risk factor for survival, with a median OS to risk ratio of 1.71 (95% CI: 1.03–2.78, p = 0.04) (70). Therefore, several therapies targeting the immune tumor microenvironment to restore antitumor immune responses have been studied in the MPM setting.

MPM has a low PD-L1 positivity rate and TPS, hence clinical trials have evaluated the antitumor activity of ICI. Though most evaluations were performed for patients with relapsed MPM after standard chemotherapy, ICIs exerted some promising results as salvage therapy (71). Three representative trials are shown below.

- KEYNOTE-028: PembrolizumabM
- This is a phase Ib study of Pembrolizumab alone in patients with PD-L1 TPS ≥1%. The efficacy was achieved with an objective response rate (ORR) of 20%, disease control rate (DCR) of 76%, median PFS of 5.8 months, and MST of 18.0 months (72).
- The NivoMes trial and MERIT: Nivolumab
- The efficacy of Nivolumab alone after second-line therapy was evaluated in a single-arm phase II study both overseas and in Japan. In the NivoMes study, conducted at a single institution in the Netherlands, ORR was 24% and DCR was 47%, median PFS was 2.6 months, 6-month survival was 74%, and MST was 11.8 months (73). On the other hand, in the MERIT trial, conducted as a multicenter trial in Japan, the ORR was 29%, the DCR was 68%, the median PFS was 6.1 months, the 6-month survival rate was 85%, and the MST was 17.3 months, showing favorable results (74).

Based on the results of MERIT, nivolumab was approved for regulatory use in patients with recurrent MPM following chemotherapy in Japan, ahead of all other countries. In addition, the antitumor effects of anti-CTLA-4 antibodies, such as ipilimumab and tremelimumab, have been evaluated in combination with nivolumab, an anti-PD-1 antibody, or durvalumab, an anti-PD-L1 antibody. Further, the possibility of ICI combination therapy has been researched. Specifically, results from three clinical trials are currently under review: NIBIT-MESO: (tremelimumab and durvalumab), MAPS-2: (ipilimumab and nivolumab combination or nivolumab monotherapy), and INITIATE: (ipilimumab and nivolumab). These studies have confirmed that combination therapy with anti-CTLA-4 and anti-PD-1/PD-L1 antibodies results in an ORR of 26 ~ 29% and a DCR of 50 ~ 68% in patients with recurrent MPM (75–77).

## Conclusion

Thus, when MPM treatment has stagnated for more than 15 years, immunotherapy with ICI is generally well tolerated and is expected to markedly improve the outcome of patients with MPM. In contrast, ICI does not necessarily provide the expected antitumor effect in all cases; the recent negative results of the PROMISE-meso trial suggest that the role of these agents should be reconsidered in pretreated MPM in unselected patients. These data should be included as they reinforce the need for biomarkers to select patients who can benefit from the treatment, especially considering the limited role of PD-L1 expression observed in this trial. The solution to these challenges lies with the investigation of reliable predictive biomarkers, which will allow the selection of appropriate candidates for improving the benefits of ICI therapy. The role of chemo-immunotherapy combinations (78) and dual blockage immune checkpoint inhibition (77) seems more promising, but the best combination of ICIs has not yet been determined. Because MPM is also characterized as an extremely inflammatory tumor, further studies focusing on MPM cells and epigenetic alterations may lead to the identification of reliable biomarkers.

## Author Contributions

KK and TM wrote the MPM treatment and Immune checkpoint inhibitors section of the manuscript. YY and MO wrote the Epigenetic alteration part of the manuscript. KK and TK provided a critical review of the manuscript. All authors contributed to the article and approved the submitted version.

## Conflict of Interest

The authors declare that the research was conducted in the absence of any commercial or financial relationships that could be construed as a potential conflict of interest.
